# Feelings of safety during daytime walking: associations with mental health, physical activity and cardiometabolic health in high vacancy, low-income neighborhoods in Detroit, Michigan

**DOI:** 10.1186/s12942-021-00271-3

**Published:** 2021-05-03

**Authors:** Amber L. Pearson, Kimberly A. Clevenger, Teresa H. Horton, Joseph C. Gardiner, Ventra Asana, Benjamin V. Dougherty, Karin A. Pfeiffer

**Affiliations:** 1 Department of Geography, Environment & Spatial Sciences, Michigan State University, East Lansing, MI USA; 2 Department of Public Health, University of Otago, Wellington, New Zealand; 3 Department of Kinesiology, Michigan State University, East Lansing, MI USA; 4 Department of Anthropology, Northwestern University, Evanston, IL USA; 5 Department of Epidemiology and Biostatistics, Michigan State University, East Lansing, MI USA; 6 Independent Scholar, Detroit, MI USA

**Keywords:** Ethnic minority, Inequality, Stress, Green space, Crime

## Abstract

**Introduction:**

Individuals living in low-income neighborhoods have disproportionately high rates of obesity, Type-2 diabetes, and cardiometabolic conditions. Perceived safety in one’s neighborhood may influence stress and physical activity, with cascading effects on cardiometabolic health.

**Methods:**

In this study, we examined relationships among feelings of safety while walking during the day and mental health [perceived stress (PSS), depression score], moderate-to-vigorous physical activity (PA), Body Mass Index (BMI), and hemoglobin A1C (A1C) in low-income, high-vacancy neighborhoods in Detroit, Michigan. We recruited 69 adults who wore accelerometers for one week and completed a survey on demographics, mental health, and neighborhood perceptions. Anthropometrics were collected and A1C was measured using A1CNow test strips. We compiled spatial data on vacant buildings and lots across the city. We fitted conventional and multilevel regression models to predict each outcome, using perceived safety during daytime walking as the independent variable of interest and individual or both individual and neighborhood-level covariates (e.g., number of vacant lots). Last, we examined trends in neighborhood features according to perceived safety.

**Results:**

In this predominantly African American sample (91%), 47% felt unsafe during daytime walking. Feelings of perceived safety significantly predicted PSS (β = − 2.34, p = 0.017), depression scores (β = − 4.22, p = 0.006), and BMI (β = − 2.87, p = 0.01), after full adjustment. For PA, we detected a significant association for sex only. For A1C we detected significant associations with blighted lots near the home. Those feeling unsafe lived in neighborhoods with higher park area and number of blighted lots.

**Conclusion:**

Future research is needed to assess a critical pathway through which neighborhood features, including vacant or poor-quality green spaces, may affect obesity—via stress reduction and concomitant effects on cardiometabolic health.

## Introduction

Individuals living in low-income neighborhoods have disproportionately high rates of obesity, Type-2 diabetes, and cardiometabolic conditions, all of which have stress- and physical activity-related etiologies [1–4]. In such neighborhoods, multiple risks operate, whereby physical activity (PA) levels are low and stress levels are high [5, 6], which leads to downstream inflammatory changes that alter body composition and metabolic and immune functions linked to chronic disease [7–10]. The theory of allostasis describes the reciprocal pathways by which the brain and body regulate myriad hormonal, neural, and immunological mechanisms by which exposure to chronic stress, including stressors resulting from neighborhood characteristics, increase risk of chronic disease across the life-span [11–13]. As part of the allostatic process of adapting to chronic stress, synaptic connections in the brain and epigenetic changes may occur. Ultimately, this may alter behavioral and endocrine readiness to respond to environmental triggers [14, 15]. Thus, it is important to recognize that perceptions of neighborhood conditions contribute to stress and influence health-related behaviors [16, 17].

Neighborhoods with poor social conditions, including high deprivation levels, crime and lack of destinations or amenities have been shown to affect stress, PA behaviors and BMI of their residents [18–21]. In addition, high levels of blighted lots or structures have been shown to affect mental health, including depression [22, 23]. Green space, in particular, has been shown to both promote and hinder crime and perceptions of safety [24–26], with potential effects on PA behaviors and stress [27–29]. Likewise, greener neighborhoods consistently predict lower obesity rates across age groups and rural/urban settings [21, 30–35]. Thus, evidence suggests that these neighborhood conditions play a role in physical health.

In addition, feelings of safety and fear of crime in one’s neighborhood have been shown to influence mental and physical health, including depressive symptoms [36] and indices of physical wellbeing [19]. Lack of perceived safety may also lead to lower levels of outdoor PA [37–40] and concomitant effects on cardiometabolic health [41]. Specifically, stress and inactivity can both lead to downstream inflammatory changes that alter body composition and metabolic and immune functions linked to chronic disease [7–10]. The primary neuroendocrine pathway conveying information about environmental stress is the hypothalamic–pituitary–adrenal axis [7, 42, 43]. This axis, in conjunction with the sympathetic and parasympathetic nervous systems [44–47], regulates many interconnected downstream pathways that alter body composition and metabolic, cardiovascular, and immune functions linked to chronic disease [7–10]. While acute stress responses may protect against threats, chronic stress and downstream inflammatory changes lead to chronic disease [42, 48, 49]. Many of these same cardiometabolic outcomes are influenced by PA [9, 10, 50–54]. High levels of chronic disease have thus been, in part, attributed to both stress and physical inactivity [55, 56].

Cardiometabolic biomarkers including glycated hemoglobin (A1C) are useful in population-based studies of chronic disease as they reveal disease risk changes well ahead of clinical disease. Elevated A1C, a marker for CVD risk and Type-2 diabetes, is a marker of chronic high blood glucose levels and insulin resistance, which can result from obesity, elevated cortisol and in response to inflammation [57–60]. Just as research shows that low PA and high stress have deleterious health effects, studies underscore the twinned health benefits whereby engaging in PA not only contributes to weight management, but also lowers stress [61]. Lower stress assists with sustained weight loss [62] and improved cardio-metabolic health [63].

Feeling safe to walk in one’s neighborhood during the day is of particular importance. Not only is walking a critical form of PA, but the daytime is when most businesses are open, and walking may be more commonly used as transportation among low-income populations. Likewise, differences in neighborhood lighting are inconsequential during the day. Yet, most studies related to perceptions of safety involve higher income neighborhoods and non-minority participants despite evidence that lower income groups may benefit more from improvements in neighborhood conditions.

Despite previous research on the mechanisms through which feelings of safety influence health, several research gaps remain. First, emerging research argues that neighborhood effects on health and behaviors may be more pronounced in low-income as compared to wealthier neighborhoods or among some subpopulations because individual-level factors interact with features of the risk environment to increase the vulnerability of individuals to such environments [64]. In fact, some studies have shown that improved neighborhood conditions (e.g., higher amounts of greenery) in low-income neighborhoods yield larger benefits, particularly for mental health [65], than those in more advantaged neighborhoods. Yet, most studies on feelings of safety have been conducted in middle- to high-income areas and in majority ethnicity samples. For example, studies of the effect of fear of crime or perceived safety have been conducted in US, New Zealand and Australian contexts among samples with high levels of education [36] and income [36, 41], majority white/European [19, 36, 39, 66] and employment [19]. Other studies omitted sample demographic information [38], including ethnicity [41]. Second, and in contrast, one study suggests that perceptions of safety are higher among African Americans, compared to other groups [39]. If consistently true, this could mean that perceptions of safety are less of a deterrent to healthy behaviors or have a smaller effect on health outcomes, compared to other groups. Therefore, exploring how feelings of safety influence health and related behaviors in low-income neighborhoods and minority populations fills a critical gap in our understanding.

Even within low-income neighborhoods, there may be micro-spatial (e.g., block-to-block) factors which influence health and related behaviors. For example, when living between two vacant homes or on a block with high vacancy, one might feel less safe about leaving home on foot. Similarly, high levels of ‘signs of disorder’ including broken windows, graffiti, unmaintained greenspaces, etc. may hinder feelings of safety for neighborhood walking. Understanding relationships between feelings of safety and health and the micro-spatial neighborhood conditions that support or hinder feelings of safety in such communities may offer critical insights to addressing pervasive socioeconomic inequalities in obesity, Type-2 diabetes, and cardiometabolic health.

The objective of this study was to examine relationships between feelings of safety during daytime walking and mental health [perceived stress score (PSS), depression score], PA behaviors and cardiometabolic health [Body Mass Index (BMI), hemoglobin A1C (A1C)] in two low-income, high-vacancy neighborhoods in Detroit, MI, USA. We hypothesize that lower perceived neighborhood safety is associated with (1) lower PA; (2) poorer mental health (stress and depression); and (3) poorer cardiometabolic health (higher BMI and A1C).

## Methods

### Sample

**Fig. 1:**
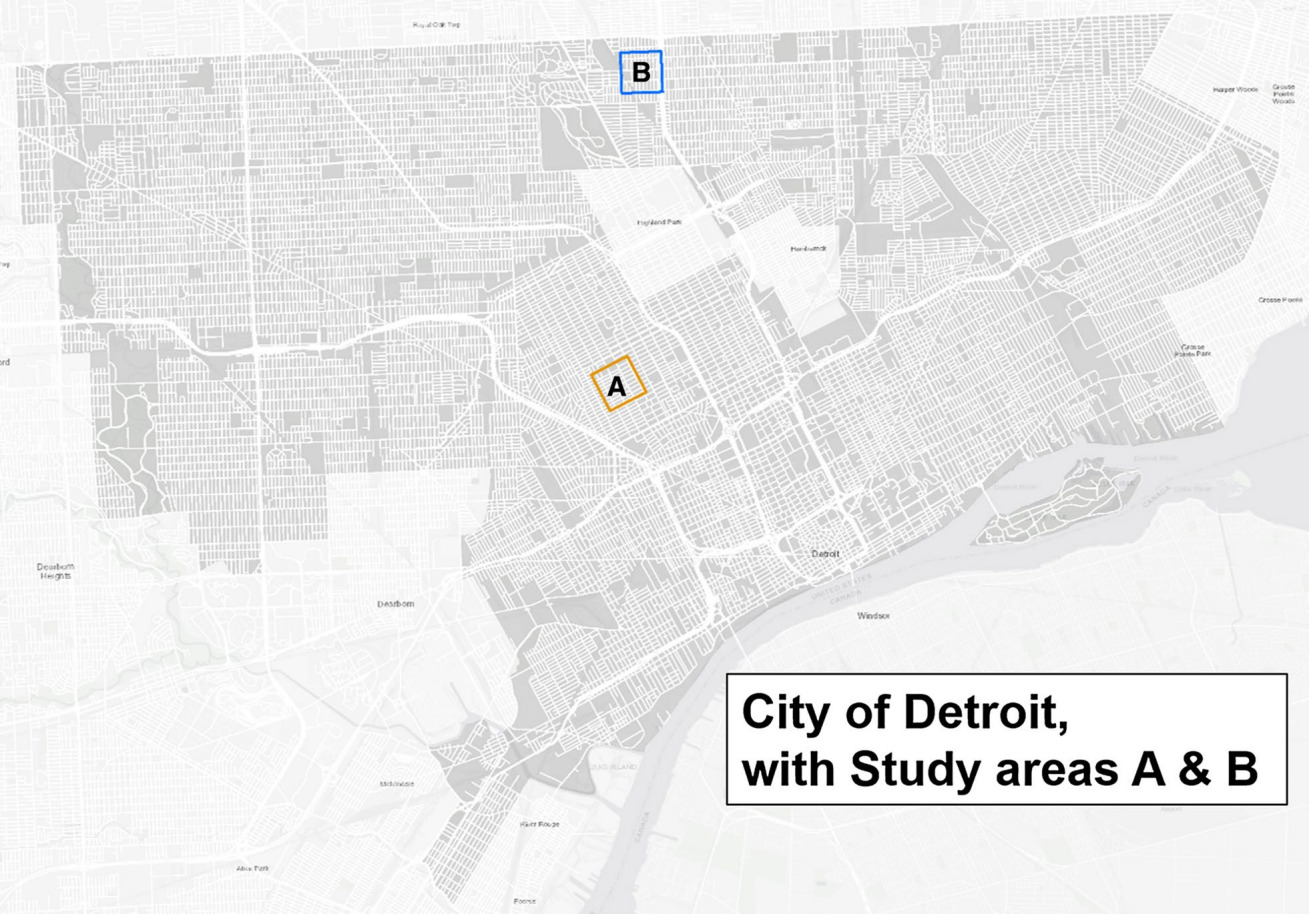
City of Detroit and the location of the two study areas

**Fig. 2:**
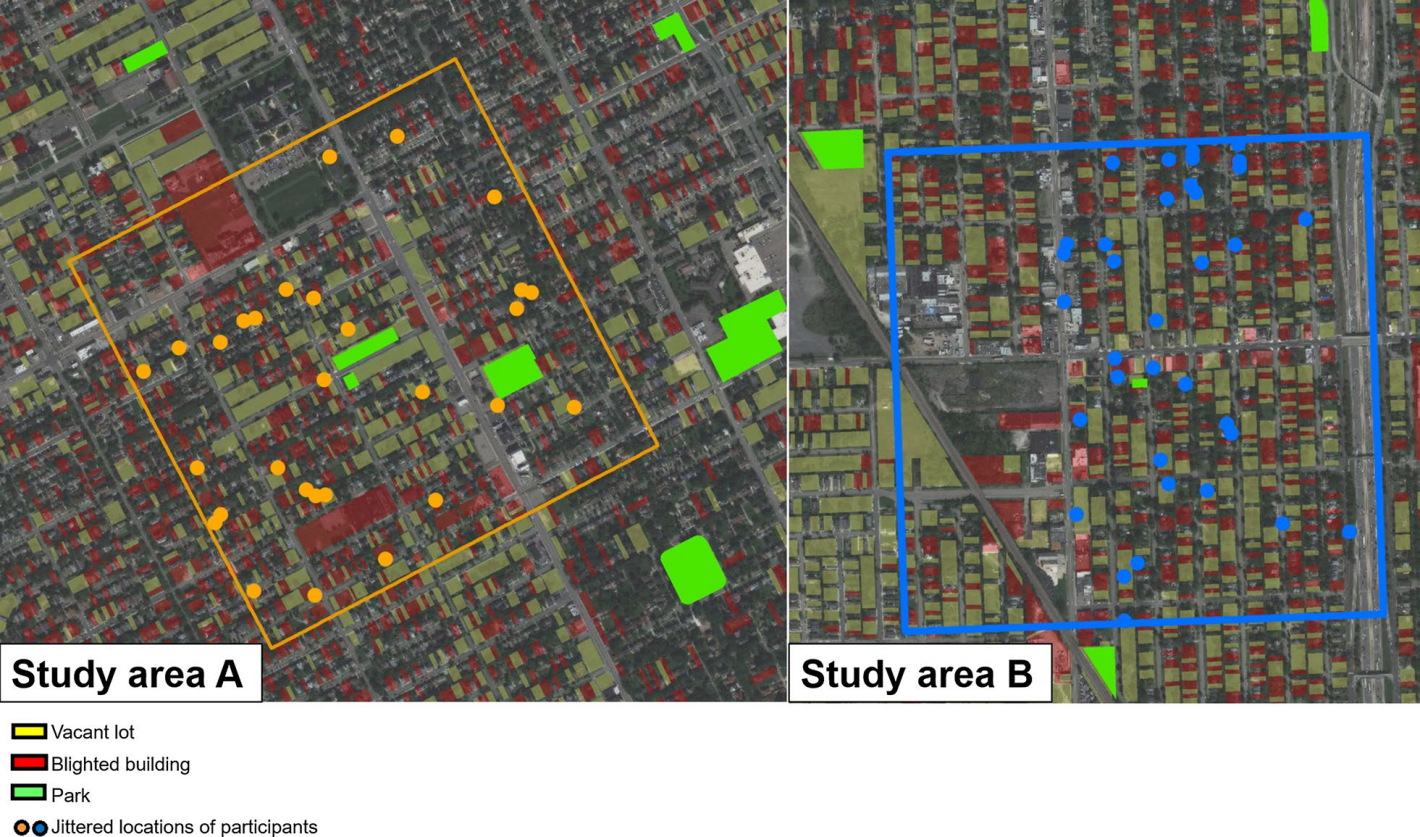
Study areas A and B, each containing high levels of vacant lots and blighted buildings and a park. Blight data republished from Data Driven Detroit under a CC BY license, with permission from Data Driven Detroit, original copyright 2013

Throughout America’s post-industrial cities, such as Detroit, severe population decline has led to high numbers of empty lots, abandoned buildings, blighted areas and unmaintained parks (as designated by the Detroit Parks and Recreation Department). We defined blighted lots as vacant lots with inactivity, presence of dumping, or lack of maintenance. We defined blighted structures as needing demolition, being vacant, having dumping, evidence of fire, or lack of security (e.g., broken windows that could be entered). From May–August 2018, we recruited 69 participants from several blocks in Detroit (IRB Approval #STUDY00000587) (Fig. 1). The study areas were selected due to their high levels of vacancy and poverty and their potential inclusion in a future community-level intervention for these reasons. Specifically, our sample intentionally included areas with unmaintained parks, as these are areas of potential change, through park restoration or community action (Fig. 2). This means that our sample may have higher proportions of African Americans, higher unemployment, vacancy and poverty than Detroit as a whole. We used a community-based sampling approach, whereby all eligible individuals within the study area were recruited for participation.

We mailed postcards to every home within the study area and staffed recruitment tables at prominent locations (e.g., in parks, in front of supermarkets) in each neighborhood, then approached households door-to-door for participation. Field staff were residents of Detroit who attended and successfully completed a one-week intensive training session on building rapport, participant recruitment, consent, use of tablets for data collection, finger stick tests, and anthropometrics. Staff were trained by a Kinesiology Ph.D. student and a senior biological Anthropologist, both authors on this study. We included only one English-speaking adult (≥ 18 years old) without mobility issues per household. We recruited as many participants as possible with the time and limited funding.

Participants who provided written consent wore an accelerometer for 1 week and completed a survey to provide demographic data: age, sex, ethnicity, employment, length residency, marital status, home ownership, household composition, as well as perceived stress, depressive symptoms, and perceptions of the neighborhood. One week later, participants attended an appointment, where anthropometrics and A1C were measured. Participants who completed perceptions of neighborhood safety and demographic survey data (n = 64) were included in descriptive analyses. Only participants who also had adequate accelerometer wear time (details below) were included in inferential analyses (n = 58).

### Perceived stress and depression scores

To measure stress, we collected perceived stress via a paper survey. The PSS is comprised of 10 items (e.g., feeling nervous) measured on a Likert-type scale, which has been validated extensively in different settings [67]. PSS scores are created by reverse scoring responses to the four positively stated items and then summing across all scale items, whereby higher scores indicated more stress (max = 40). Using methods from previous research [68], we imputed missing items within the PSS by carrying forward the response from the previous item when fewer than four responses were missing. Items 4, 5, 7 and 8 were first reverse coded (positively stated items). So, for example, when item 3 was missing, the response for item 2 was be used. When item 1 was missing, the response for item 10 was used. We used the imputed data for all analyses, as we detected < 10% difference in coefficients on the non-imputed versus imputed data. In our sample, the Cronbach’s α (a measure of internal consistency, often used for indices or scales) for PSS was greater than 0.7 and therefore deemed acceptable or good (0.77). The perceived stress scale’s face validity and scale content were ranked high with a Kaiser–Meyer–Olkin coefficient (a measure of how well the data are suited for factor analysis) of 0.82 [69]. The scale’s internal consistency reliability was good in multiple languages and convergent validity was supported by expected relationships with other mental health measures, including anxiety and depression [70]. In our sample, scores ranged from 4 to 34.

*Depressive symptoms* were collected via NIH’s Adult PROMIS-29 Profile v2.0 [71, 72], and t-scores were generated by comparing values to the online tool reference population, the 2000 general US census population (mean of 50, standard deviation of 10). Lower t-scores represent more favorable outcomes. PROMIS measures have been shown to significantly change in diverse clinical samples, following interventions likely to influence negative affect [73]. The PROMIS-29’s internal consistency for sub-domains has been shown to be high (Cronbach’s α > 0.88), with adequate structural validity for most domains (CFI > 0.95, RMSEA < 0.05, both metrics are model fit indices) [74]. In our sample, the Cronbach’s α for depression variables was excellent (0.95). In our sample, scores ranged from 41 to 79.

### Physical activity levels

Participants wore a triaxial accelerometer on an elastic belt around their waist, positioned over the right hip, during waking hours for 1 week. Due to limited funding, two accelerometer models were used (ActiGraph GT3X and wGT3X-BT), which measured acceleration at a sampling rate of 30 Hz to generate activity counts/60-s. Published research suggests comparability between accelerometer models [75]. Non-wear time was defined as 60 min of continuous zeros and at least four days, of at least 480 min of valid wear data, were required for inclusion in subsequent analysis [76, 77]. Minutes/day of moderate-to-vigorous PA (MVPA) were determined using the Freedson et al. [78] cut-points, which is the most widely used classification scheme for accelerometer-measured PA in adults [77].

### A1C and BMI

A1C [16, 60], which is an indicator of blood glucose levels over the prior 3-months and is often used to test for prediabetes, was measured from blood samples collected from finger-tip sticks using portable analyzers and test strips (A1CNow^+^®, PTS Diagnostics), previously shown to be valid compared to lab-based analysis [79]. Stature was measured using a portable stadiometer (SECA Corp) and mass was measured using a digital scale (Tanita). Two measurements were taken and averaged. BMI values were calculated as kg/m^2^.

### Perceptions of neighborhood daytime safety

Perceived neighborhood safety was assessed as whether or not a participant feels safe walking during the day [80]. We asked: “The crime rate in my neighborhood makes it unsafe to go on walks during the day.” Responses were recorded on a Likert-type scale for level of agreement. These were aggregated to three levels, where 1 = “Strongly Agree” or “Agree a Little”; 2 = “Neither Agree nor Disagree”; and 3 = “Strongly Disagree” or “Disagree a Little”. The purpose of walking was not specified in the question (i.e., for leisure or for transportation). Questions about the perceptions of the neighborhood [80–84] such as feelings of safety [19], have been shown to have moderate to high agreement or correlation (rho range = 0.42–0.91) [81].

### Area-level characteristics

Consistent with theoretical area-level drivers of both mental health and cardiometabolic health, we also compiled additional area-level characteristics for correlation analyses including deprivation, crime, blighted lots or structures, and green space. In this study, we included a crime index (both property and violent crime). Each participant was assigned a value based on their home location’s census block. This index is part of the Neighborhood Change Index created by Data Driven Detroit (D^3^) in October 2018, for the ‘Turning the Corner’ project using 2010 census blocks [85]. The following Detroit datasets were used by D^3^: City of Detroit Building, Safety Engineering and Environmental Department, Department of Administrative Hearings, Data Driven Detroit’s Rental Property Analysis, Fire Department, Police Department, Water and Sewerage Department, DTE Energy, and Property Praxis [85]. D^3^ excluded: (1) blocks with five or fewer residential structures according to a 2014 in-person audit; (2) blocks where fewer than 25% of parcels had a residential structure; and (3) blocks where the median home value exceeds $150,000 using the American Community Survey, 2011–2015. With all remaining blocks, D^3^ generated z-scores for each variable at the block level. After factor analysis, only those with heavy loadings (> 0.45) were kept. Z-scores of relevant indicators were averaged to create index variables. Index values range from 1 (low) to 5 (high).

We also included the number of blighted lots and blighted structures within a 100 m Euclidean (as the crow flies) buffer around each participant’s home, based on 2014 data from in-person audits (Motor City Mapping Project [86]). We also calculated the area of each buffer covered by a park, using data from the Detroit Parks and Recreation Department. Last, we assigned each participant the area deprivation rank score based on their census block group, calculated from 2013 census data and compiled by the University of Wisconsin [87]. All spatial techniques were conducted using ArcMap v10.6 (ESRI, Redlands, CA).

### Statistical analyses

For continuous and categorical variables, we calculated averages or percentage values for the whole sample and stratified by level of perceived safety during daytime walking. We also tested for significant differences in demographic and health measures by level of feelings of safety. For binary variables, we calculated p-values using chi-square exact test; for continuous variables, we calculated p-values using Wilcoxon test.

Next, for each outcome separately, we fitted two linear models. All outcome variables (perceived stress scores, depression t-scores, BMI, A1C and square root of MVPA) were all treated as continuous variables. The square root transformation was applied to MVPA to mitigate skewness. The independent variable of interest, feelings of safety during daytime walking, was treated as an ordinal variable. The *first model* included individual-level covariates (age was treated as a continuous variable; sex and employment were treated as binary variables). The *second model* included both individual and area-level covariates (blighted lots was treated as a continuous variable; crime index was treated as an ordinal variable). A random effect for block level is included in all models to capture possible correlation between subjects within block. ICC (intra-class correlation) could be viewed as a correlation in outcomes between subjects in the same block. None of ICCs of the null models for the five outcomes were statistically significant, except for A1C (ICC = 0.657). Last, we examined neighborhood features according to level of perceived safety during daytime walking and used a Spearman’s correlation coefficient (rho) to evaluate relationships between feelings of safety and neighborhood characteristics due the ordinal nature of feelings of safety. All statistical analyses were conducted using Stata v16 (Statacorp, College Station, TX) and SAS Software ver 9.4 (SAS Institute Inc, Cary, NC).

## Results

On average, participants who felt unsafe walking during the day were slightly older, had fewer children, and lived in their neighborhood for less time (Table 1). Generally, African–Americans (91% of our sample), employed participants and single people more often reported feeling unsafe walking during the day. In contrast, among those that felt safe walking during the day, 63% were female, 80% were African American, 47% were employed, 20% were married or partnered, and 43% owned their home. We also observed a clear gradient between feelings of safety and perceived stress scores and lower depressive scores among those feeling safe walking. We observed higher levels of PA among those feeling unsafe, but this group also had higher BMI and A1C. We also observed a trend toward higher crime, more blighted lots and buildings, and higher park area among those we felt unsafe walking during the day. The only significant differences across levels of feelings of safety were found for African Americans (p = 0.036) and depressive symptoms (p = 0.006).

**Table 1:** Sample characteristics, stratified by level of perceived safety during daytime walking and for the total sample

	Feel unsafe walking	Neutral	Feel safe walking	Total in our sample	Blockgroup data for our study area^a^	Blockgroup data for Detroit^a^
N	24	10	30	64	6456	1,339,576
Female, %	45.8	90	63.3	60.9	69.9	68.5
African–American, %	100	100	80	90.6	86.7	47.5
Employed, %	60.9	44.4	46.7	51.6	38.5	53
Married or partnered, %	12.5	11.1	20	15.9	5.1^b^	13.2^b^
Own home, %	34.8	11.1	42.9	35	56.8	62.1
Number children, mean (sd)	1.0 (1.4)	1.9 (3.0)	0.5 (0.9)	0.9 (1.6)	0.3 (0.2)	0.3 (0.2)
Age, mean years (sd)	44.7 (13.9)	43.4 (18.0)	42.9 (15.9)	43.7 (15.3)		
Length residency, mean years (sd)	4.1 (4.4)	5.0 (2.8)	8.6 (11.3)	6.3 (8.4)		
Perceived stress score, mean (sd)	19.3 (6.9)	18.8 (5.2)	15.2 (6.1)	17.3 (6.5)		
Depressive symptoms, mean (sd)	56.7 (11.6)	57.9 (9.0)	48.3 (9.0)	52.9 (10.8)		
MVPA, min/day mean (sd)	28.1 (22.5)	18.2 (11.4)	19.5 (22.7)	22.8 (21.4)		
BMI, kg/m^2^ mean (sd)	32.8 (8.5)	30.4 (6.1)	29.0 (6.8)	30.7 (7.5)		
A1C, % mean (sd)	5.8 (1.5)	5.4 (1.4)	5.4 (0.7)	5.5 (1.2)		
Crime index^c^, mean (sd)	3.3 (1.3)	3.2 (1.5)	3.1 (1.5)	3.2 (1.4)		
Blighted lots, mean (sd)	22.4 (10.8)	18.5 (11.2)	15.6 (9.7)	18.6 (10.7)		
Blighted buildings, mean (sd)	18.1 (7.3)	17.8 (10.1)	17.7 (8.3)	17.9 (8.1)		
Area of park in km, mean (sd)	0.7 (1.6)	0.2 (0.6)	0.3 (1.1)	0.4 (1.3)		

*MVPA* moderate-to-vigorous intensity physical activity, *BMI* body mass index, *A1C* glycated hemoglobin

^a^Blockgroup data from 2015 to 2019 ACS data. Source: [113]

^b^Census data do not include partnered adults

^c^Quintiles from high (5) to low (1)

Compared to the blockgroups from which our sample was taken, our sample had slightly lower percentage females, home ownership and number of children, slightly higher percentage African Americans, higher employment and married percentages (although census data do not include partnered adults). Compared to Detroit as a whole, our sample had slightly lower percentage females, home ownership and number of children, and higher percentage African Americans.

Feelings of safety during daytime walking was significantly negatively associated with both PSS and depressive symptoms, regardless of level of confounder control (Table 2, Models A–D). When feelings of safety were higher, PSS and depressive symptoms were significantly lower. As seen in Fig. 1, the study areas have high numbers of blighted, vacant lots and there is high variation from one block to another. Despite this variation, the number of blighted lots or crime index values did not significantly influence mental health measures, independent of feelings of safety while walking (Table 2, Models B and D). In fact, when neighborhood confounders were added to individual-level confounders, the absolute value of the coefficient (β) increased for PSS. No other covariates showed significant, independent effects.

**Table 2:**
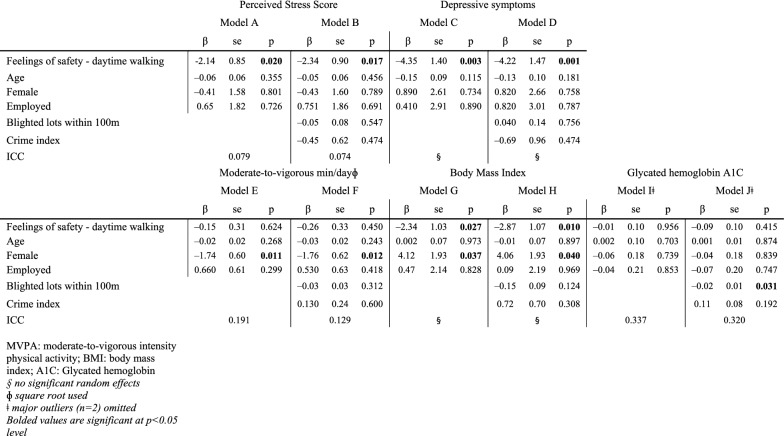
Regression modelling results, for Perceived Stress Scores (Models A & B), depressive symptoms (Models C & D), physical activity (Models E & F), body mass index (Models G & H), and glycated hemoglobin A1C (Models I & J)

*MVPA* moderate-to-vigorous intensity physical activity, *BMI* body mass index, *A1C* glycated hemoglobin

Bold italic values are significant at p < 0.05 level

^a^No significant random effects

^b^Square root used

^c^Major outliers (n = 2) omitted

For PA, the only significant predictor was sex (Table 2), whereby we would expect around 19.3 more minutes of moderate-to-vigorous PA per day for males compared to females, after accounting for all covariates (Model F). For BMI, we observed significant, negative associations with perceived safety during daytime walking (Table 2, Models G and H). In the fully adjusted model, we would expect 2.9 lower BMI for every one-point increase in perceived safety. Female sex was also significantly associated with higher BMI. For A1C, we observed an unexpected negative association with the number of blighted lots within 100 m (Table 2, Model J).

In terms of area-level factors of perceived safety during daytime walking, most correlations were weak (Table 3). We observed the largest correlations for blighted lots, area deprivation and park area, respectively. The average number of blighted lots near those feeling unsafe was 22.4, compared to 15.6 for those feeling safe. Participants’ feelings of safety and the number of blighted lots near their home was significantly correlated (rho = − 0.288, p = 0.022). Participants’ feelings of safety was weakly, negatively correlated with the level of area deprivation (rho = − 0.148, p = 0.246), although not statistically significant. Also, participants’ feelings of safety was negatively, weakly correlated with the extent of park area near their home (rho = − 0.135, p = 0.295), although again not statistically significant. Mean crime index scores were slightly lower in areas where residents reported feeling safe, but the correlation was non-significant.

**Table 3:** Trends in neighborhood characteristics according to participants’ perceived safety during daytime walking, including ratios and Spearman’s correlations

	Feel unsafe walking [1]	Neutral [2]	Feel safe walking [3]	Total	Spearman’s rho	Spearman’s p-value
Area deprivation, mean	9.6	9.5	9.4	9.5	− 0.148	0.246
Blighted lots, mean	22.4	18.5	15.6	18.6	− **0.288**	**0.022**
Blighted structures, mean	18.1	17.8	17.7	17.9	− 0.042	0.746
Crime index^a^, mean	3.3	3.2	3.1	3.2	− 0.050	0.700
Park area in m^2^, mean	696.6	198.8	265.9	421.8	− 0.135	0.295

Bolded font p ≤ 0.05

^a^Crime index: 5 = more crimes, 1 = fewer crimes

## Discussion and conclusion

Overall, in this predominantly African American sample, 47% felt unsafe during daytime walking. We did not confirm our hypothesis that lower perceived neighborhood safety is associated with lower PA, as the only significant variable was sex. Our second hypothesis that lower perceived neighborhood safety would be associated with poorer mental health (stress and depression) was confirmed after full adjustment of covariates. We partially confirmed our third hypothesis that lower perceived neighborhood safety would be associated with poorer cardiometabolic health; only higher BMI, but not A1C, was related (after full adjustment for covariates). Additionally, those feeling unsafe lived in neighborhoods with higher park area and number of blighted lots. Thus, findings were mixed compared to the expected nature of the relationships examined.

It is important to consider that nearly half of the sample did not report feeling safe walking in their neighborhood during the daytime. Because walking is an important form of exercise [88] and walking near home is accessible to those without a vehicle or is needed to take advantage of public transportation, any obstacles to this form of exercise are highly important for cardiometabolic health in low-income populations. Our study points to one important obstacle to engaging in walking in one’s neighborhood: feelings of safety. Here, we discuss our study findings, drawing comparisons to other studies in minority ethnicity, low-income samples when possible. Still, direct comparisons with such studies or studies among high-income and/or majority ethnicity samples are constrained by differences in measures of perceptions of safety, objective versus self-reported measures, and differences in health outcome measures.

### Perceived safety and mental health

We found that lower feelings of safety were associated with higher stress, depression, and BMI. In contrast, feelings of safety were not associated with PA levels or A1C. These findings suggest that perceptions of safety in one’s neighborhood may have a significant influence on mental health and obesity, even after accounting for recorded crime. Other research from New Zealand has shown that perceptions of safety can be quite spatially focal, whereby fear of crime was associated with recorded crime in one’s own neighborhood, but not in surrounding neighborhoods [89] and that the relationship between recorded crime and fear is moderated by neighborhood social context [90]. This work also found that perceptions of lower safety were associated with poorer mental and physical outcomes, yet recorded crime had little or no independent effect on health [19, 91], echoing our findings. Our findings are also in line with a growing body of evidence that shows that lack of perceived safety can negatively affect anxiety [92, 93], psychological distress [94, 95], mental wellbeing [19] and depressive symptoms [36]. Interestingly, one study found that the association between higher perceived safety was associated with better mental health outcomes was mediated by PA [96]. A caveat to this study is that perceived neighborhood safety was determined by 30 university volunteers using imagery of neighborhoods, rather than the participants for which outcomes were measured.

No other demographic covariates were independently associated with mental health after accounting for perceived safety and neighborhood conditions. This is unexpected given the breadth of research showing that, in studies of perceptions of safety, mental health tend to vary by sex, age, marital status and other demographic characteristics (e.g., [19]). Our findings highlight a need for a more nuanced understanding of how perceptions of safety arise in some groups but not in others, potentially including past experiences or victimization, or social position.

### Perceived safety, physical activity and cardiometabolic outcomes

Somewhat surprisingly, we found that feelings of safety were associated with BMI, but not PA levels, in contrast to some existing work [66]. A meta-analysis of the relationship between perceived safety and PA concluded that those reporting feeling safe had a 27% greater odds of having higher levels of PA (OR = 1.27, 95% CI = 1.08, 1.49), although effects were heterogeneous [97]. One possible explanation for our incongruent finding is that the neighborhood-obesity relationship is influenced not only by PA, but through the stress pathway [7–10]. Other studies of neighborhood conditions have also found associations with BMI but not PA. For example, many studies on neighborhood green space and health have detected significant associations with lower BMI but no association with PA [21, 30–35, 98]. Another study among socioeconomically and racially/ethnically diverse adolescents in Minneapolis found that BMI, but not PA, was positively associated with perceived crime [99]. BMI was also positively associated with lack of perceived safety in a majority Latino sample in Los Angeles [100]. In a recent study in a predominantly African American (93%) sample, the opposite was found [101]. Specifically, perceived improvements in neighborhood safety over a 3-year period were associated with significantly higher BMI, particularly among females, but not after adjustment for baseline BMI. A second possible reason for our findings is that participants in our sample did not have adequate variation in PA levels. Overall, our sample was rather sedentary, with an average of only 23 min of MVPA per day, compared to a USA average of approximately 35 min/day for males and 20 min/day for females [88].

Unexpectedly, we also found a negative association between A1C and number of blighted lots. Echoing our unexpected findings, another study comprised primarily of African Americans found a positive association between A1C and perceived improvements in neighborhood safety among females only [101]. However, this relationship attenuated to non-significance after adjustment for baseline BMI. Still, these findings are surprising because qualitative research from Detroit has shown that landscape maintenance is a visible sign of care which contributes to changes in physical and social environments linked to health [102]. In that study, signs of care (e.g., mowing vacant lots) strengthened social relationships among neighbors, lowered stress and offered a coping strategy to handle stress effects. Another study had similar results in a majority African American sample, finding that percent vacancy was positively associated with fear of walking [37]. One possible explanation for our finding is that areas where demolitions are high may be areas where there is more activism to get abandoned buildings removed, thus higher numbers of vacant lots. Since we also observed higher numbers of blighted lots in areas where participants felt more unsafe, there are likely unmeasured confounders affecting our evaluation of the relationship between A1C and feelings of safety.

### Practical implications

Understanding the area-level conditions of those feeling unsafe may provide some insights as to potential points of intervention for improving perceived safety and associated outcomes, like perceived stress, depressive symptoms, and BMI. Those feeling unsafe lived in areas with a higher average park area and number of blighted lots. Perhaps more importantly, given our findings, unmaintained green spaces in low-income, high vacancy areas may confer negative effects. While maintained green spaces have been shown to promote PA and lower stress [29, 103–105], unmaintained spaces (both as parks and as vacant lots) may not yield benefits. In the worst instances, these areas become locations for drug dealing and crime in the U.S. [106]. It is important to note that in other countries unmaintained spaces may be viewed and utilized differently than those in the U.S. On the positive side, experimental studies have shown that improving poorly maintained green spaces and/or ‘greening’ vacant lots leads to physical health benefits, lower stress, lower neighborhood violence, and increased perceived safety [26, 107, 108].

So, while our study showed that parks and blighted lots were more common in areas with lower perceived safety, improvements in these neighborhood features offer the promise of improved perceived safety and also health conditions. Still, beyond identifying neighborhood features that are obstacles to healthy living, future research could usefully, in countries and areas that warrant it, utilize a structural violence framework to assessing PA behaviors and cardiometabolic health, given the high levels of stress experienced in such low-income neighborhoods. Such research could examine how historical processes such as redlining, housing exclusion policies and employment opportunities create inequitable environments that influence individuals. Specifically related to fear and lack of safety, research has shown that fear, created by structural forces, is a driving factor undermining diabetes and healthcare behaviors among Hispanic communities [109]. Perhaps a similar framework can be used in future research to understand historical structural and cultural factors which produce or exacerbate fear in one’s neighborhood, as this relates explicitly to PA behaviors.

### Study limitations

This study has limitations to consider. The blight data were collected in 2014 and conditions may have changed. We measured total PA, rather than PA that occurred within a certain distance of their home location. It is possible that PA may have occurred in other settings: potentially less disadvantaged areas. This may, in part, explain the lack of a detected association between PA and feelings of safety. Future research could restrict analysis to only PA occurring within a certain distance from the home. Mental health measures were self-reported and it is unclear how these measures might compare to objective measures of stress (e.g., salivary cortisol). The sample size was limited in this study. For this reason, we were unable to explore the potential for the relationship between perceptions of safety and BMI to be mediated by PA. Future research with a larger sample may contribute to our knowledge with such analyses.

Another limitation of this study is the lack of data collection on diet, due to the additional burden on participants, which is also likely important for assessing drivers of BMI. We also did not measure differential access to healthy food options in the built environment. Indeed, PA alone may not be sufficient to offset the effects of poor diet [110]. Given the inconsistency in findings in the literature between neighborhood conditions and A1C [111, 112] and in the measurement of neighborhood safety, our non-significant but positive findings warrant further exploration in future research.

### Future research

Future research is also needed to assess a critical pathway through which area-level conditions, particularly unmaintained parks and blighted vacant lots, affect obesity via stress reduction and concomitant effects on cardiometabolic health. It is unclear whether stress reduction alone leads to improved cardiometabolic health (including lower BMI) or whether PA is the precursor for stress reduction and the cascading health benefits. Correlational designs do not answer this question, as it is entirely possible that people with lower BMI or better cardiometabolic health choose to live in particular settings. Qualitative study designs may help shed light on some of the surprising findings or help untangle the ways in which perceptions of safety, personal characteristics and neighborhood conditions interact to influence health and related behaviors. Additionally, qualitative studies can shed light on how individuals from countries outside of the U.S. view neighborhood safety and characteristics compared to individuals in the U.S. Experimental or quasi-experimental designs are needed to untangle these potential neighborhood effects on behavior, weight status and downstream cardiometabolic health, particularly in low-income neighborhoods. Importantly, inexpensive park and vacant lot interventions are needed to assess causality. In low-income neighborhoods, like those studied in Detroit, such interventions are more feasible than typical park projects that install costly equipment and trail systems.

### Conclusions

While it may be a challenge to eliminate chronic disease and stress, particularly in disadvantaged settings, it is the hope that promotion of PA and stress reduction may also help in the management of existing chronic diseases or stress reduction. Community-level interventions, such as neighborhood improvements that bolster safety perceptions, may offer one avenue for equitable promotion of stress reduction, and lower BMI and depression in disadvantaged neighborhoods, as our findings suggest. Still, future research is needed to assess a critical pathway through which neighborhood features, including vacant or poor-quality green spaces, may affect obesity—via stress reduction and concomitant effects on cardiometabolic health.

## Data Availability

The datasets during and/or analysed during the current study available from the corresponding author on reasonable request.
